# L-Tryptophan Production in *Escherichia coli* Improved by Weakening the Pta-AckA Pathway

**DOI:** 10.1371/journal.pone.0158200

**Published:** 2016-06-27

**Authors:** Lina Liu, Xuguo Duan, Jing Wu

**Affiliations:** 1 State Key Laboratory of Food Science and Technology, Jiangnan University, 1800 Lihu Avenue, Wuxi, 214122, China; 2 School of Biotechnology and Key Laboratory of Industrial Biotechnology Ministry of Education, Jiangnan University, 1800 Lihu Avenue, Wuxi, 214122, China; University of Houston, UNITED STATES

## Abstract

Acetate accumulation during the fermentation process of *Escherichia coli* FB-04, an L-tryptophan production strain, is detrimental to L-tryptophan production. In an initial attempt to reduce acetate formation, the phosphate acetyltransferase gene (*pta*) from *E*. *coli* FB-04 was deleted, forming strain FB-04(*Δpta*). Unfortunately, FB-04(*Δpta*) exhibited a growth defect. Therefore, *pta* was replaced with a *pta* variant (*pta*1) from *E*. *coli* CCTCC M 2016009, forming strain FB-04(*pta*1). Pta1 exhibits lower catalytic capacity and substrate affinity than Pta because of a single amino acid substitution (Pro69Leu). FB-04(*pta*1) lacked the growth defect of FB-04(*Δpta*) and showed improved fermentation performance. Strain FB-04(*pta*1) showed a 91% increase in L-tryptophan yield in flask fermentation experiments, while acetate production decreased by 35%, compared with its parent FB-04. Throughout the fed-batch fermentation process, acetate accumulation by FB-04(*pta*1) was slower than that by FB-04. The final L-tryptophan titer of FB-04(*pta*1) reached 44.0 g/L, representing a 15% increase over that of FB-04. Metabolomics analysis showed that the *pta*1 genomic substitution slightly decreased carbon flux through glycolysis and significantly increased carbon fluxes through the pentose phosphate and common aromatic pathways. These results indicate that this strategy enhances L-tryptophan production and decreases acetate accumulation during the L-tryptophan fermentation process.

## Introduction

L-tryptophan, an essential amino acid that is also a precursor of other important biomolecules, such as the neurotransmitter serotonin, is widely used in medicine, food, and animal feed [1, 2]. The L-tryptophan biosynthetic pathway in microorganisms, which involves central metabolism, the common aromatic pathway and the L-tryptophan branch pathway, is long and its regulation is complicated [3–5]. The high industrial relevance of *Escherichia coli* has stimulated efforts to improve L-tryptophan yield by analyzing the underlying metabolic regulatory networks of L-tryptophan biosynthesis and altering them through targeted modifications [6–8]. Despite substantial effort, by-product accumulation continues to limit L-tryptophan production.

The accumulation of acetate, a main by-product of fermentation process, can slow cell growth [9, 10]. In *E*. *coli*, two metabolic pathways are involved in acetate formation: the PoxB oxidase pathway and the Pta-AckA pathway [11]. In the PoxB oxidase pathway, pyruvate oxidase (PoxB; EC 1.2.5.1), encoded by the *poxB* gene, catalyzes the formation of acetate [11]. In the Pta-AckA pathway, which is the primary acetate formation pathway, phosphate acetyltransferase (Pta; EC 2.3.1.8) and acetate kinase (AckA; EC 2.7.2.1), encoded by the *pta* and *ackA* genes, respectively, catalyze the formation of acetate from acetyl-CoA in two successive steps [12, 13]. Pta, the key enzyme in this pathway, catalyzes the conversion of acetyl-CoA to acetyl phosphate, which plays an important cellular role as a phosphorus donor [14]. Knockout of *pta* is the most common genetic manipulation used to diminish acetate accumulation. Although a *pta* deletion mutant has shown increased biomass and a higher capacity for producing L-tryptophan, compared with its parental strain [15], most previous reports indicate that Pta has a significant physiological function in *E*. *coli*, and that deletion of *pta* adversely impacts cell growth [14, 16–18]. Remarkably, no study examining the effect of *pta* mutation, rather than deletion, on acetate formation, has been reported, even though this strategy may decrease acetate formation without the deleterious effects of a *pta* knockout.

Recombinant *E*. *coli* FB-04 was constructed in our laboratory for the production of L-tryptophan [5]. However, problems with acetate accumulation during the fermentation process hinder its usefulness. In the initial phase of this study, the native *pta* in the FB-04 genome was knocked out, forming strain FB-04(*Δpta*). This knockout led to a growth defect. Then, the *pta* in the FB-04 genome was replaced with the mutant *pta*1 identified from *E*. *coli* CCTCC M 2016009, which accumulates less acetate during fermentation process, to form the strain FB-04(*pta*1). This substitution not only reversed the growth defect caused by *pta* deletion, it also diminished acetate accumulation and improved L-tryptophan production in FB-04(*pta*1). For the sake of completeness, the performance of FB-04(*pta*1) was also compared with that of FB-04(*ΔackA*), an AckA deletion mutant. Metabolomics analysis, a powerful method that has proven to be an important tool for the analysis of changes in intracellular metabolite levels [19, 20], was used to investigate the metabolic distinctions among the L-tryptophan production strains.

## Materials and Methods

### Strains, plasmids, and genetic methods

The background, genotypes, and sources of all strains used or produced in this study, along with the primers and plasmids used in their construction, are listed in Table 1. *E*. *coli* strains JM109 and BL21(DE3), which were used for plasmid construction and protein expression, respectively, were obtained from Novagen (Madison, USA). *E*. *coli* CCTCC M 2016009 was isolated from the soil at Kanas Lake, Xinjiang Uygur Autonomous Region, China (48°43’N 87°01’E) and stored in our laboratory. *Bacillus subtilis* ATCC 6051a, obtained from the American Type Culture Collection (ATCC), supplied the genomic DNA used during the construction of the plasmid pMD-SK. Recombinant *E*. *coli* FB-04 was previously constructed for L-tryptophan production [5]. *E*. *coli* strains FB-04(*Δpta*), FB-04(*ΔackA*) and FB-04(*pta*1) are derivatives of FB-04 (Table 1). The tool plasmids pKD13 and pKD46 used for gene knockout procedures were purchased from the *E*. *coli* Genetic Stock Center (Yale University, New Haven, USA). The pMD18-T simple vector and pET24a vector were obtained from Takara (Dalian, China).

**Table 1:** Strains, plasmids and primers used in this study.

Strains/plasmids/primer	Description/Genotype/Sequence	source
**Strains**		
FB-04	*E*. *coli* W3110 k12, *ΔtrpR*::FRT*ΔtnaA*::FRT*ΔpheA*::FRT	[5]
	*ΔtyrA*::FRT, harboring pSTV-03 plasmid	
*B*. *subtilis* ATCC 6051a	Providing template for *sacB*	ATCC
*E*.*coli* CCTCC M 2016009	Isolated from the soil	CCTCC
BL(DE3)	*E*. *coli* host for protein expression	Novagen
BL21(DE3)/pET24a-*pta*	BL21(DE3) harboring pET24a-*pta*	This study
BL21(DE3)/pET24a-*pta*1	BL21(DE3) harboring pET24a-*pta*1	This study
FB-04(*Δpta*)	*Δpta*::*kan-sacB*; *pta* deletion mutant derived from strain FB-04	This study
FB-04(*ΔackA)*	*ΔackA*::*kan-sacB*; *ackA* deletion mutant derived from strain FB-04	This study
FB-04(*pta*1)	*pta*1 genomic substitution in FB-04	This study
**Plasmids**		
pKD13	Amp and kan markers	[21]
pKD46	Amp markers, helper plasmid	[21]
pSTV-03	Based on plasmids of pACYC177 and pND707; p15A replicon, kan	[5]
	marker, PR and PL promoters, carrying *aroF*^*fbr*^ and *trpE*^*fbr*^*D*	
pET24a	Protein expression vector in *E*. *coli* BL(DE3)	Novagen
pET24a-*pta*	pET24a harboring *pta*	This study
pET24a-*pta*1	pET24a harboring *pta*1	This study
pMD18-T	Cloning vector	Novagen
pMD-SK	pMD18-T harboring *kan* and *sacB*	This study
**Primers**^**a**^		
C-*pta*	5’-aaacatatgtcccgtattattatgctgatcc-3’	This study
	5’-cccaagcttttactgctgctgtgcagactg-3’	
P-*pta*	5’-gtgtcccgtattattatgctgatccctaccggaaccagcgtcggtctgac	This study
	attccggggatccgtcgacc-3’	
	5-ttactgctgctgtgcagactgaatcgcagtcagcgcgatggtgtagacga	
	tgccaataggatatcggcat-3’	
P-*ackA*	5’-aggtacttccatgtcgagtaagttagtactggttctgaactgcggtagtt	This study
	attccggggatccgtcgacc-3’	
	5-accgccagctgagctggcggtgtgaaatcaggcagtcaggcggctcgcgt	
	tgccaataggatatcggcat-3’	
V-*ackA*	5’-atgtcgagtaagttagtact-3’	This study
	5’-tcaggcagtcaggcggctcgcgt-3’	
V-*pta*	5’-aagcggctttaggtgcaggc-3’	This study
	5’-tttactgctgctgtgcagactgaat-3’	
V-*poxB*	5’-atgaaacaaacggttgcagc-3-3’	This study
	5’-ttaccttagccagtttgtt-3’	
SK-*kan-sacB*	F:5’-aaacatatgattccggggatccgtcgacc-3’	This study
	R:5’-cccaagctttgccaataggatatcggcat-3’	
	Overlap-F:5’-cgaagcagctccagcctacagcaactttatgcccatgca-3’	
	Overlap-R:5’-tgcatgggcataaagttgctctaggctggagctgcttcg-3’	

The sequences of primers V-*pta*, V-*ackA*, and V-*poxB*, which were designed for PCR amplification of the genes *pta* (GenBank accession number gi 946778), *ackA* (GenBank accession number gi 946775), and *poxB* (GenBank accession number gi 946132), respectively, are shown in Table 1. These primers were used to amplify the genes *pta*, *ackA* and *poxB* using genomic DNA from *E*. *coli* FB-04 as the template. They were also used to amplify the genes *pta*1, *ackA*1, and *poxB*1 using genomic DNA from *E*. *coli* CCTCC M 2016009 as the template. To express Pta and its mutant Pta1, the PCR products *pta* and *pta*1, obtained from *E*. *coli* FB-04 and CCTCC M 2016009, respectively, using the primer set C-*pta* (Table 1), were digested with *Nde*I and *Hin*dIII, and then ligated into plasmid vector pET24a to form plasmids pET24a-*pta* and pET24a-*pta*1. These two plasmids were used to transform the expression strain *E*. *coli* BL21(DE3), resulting in strains BL21(DE3)/pET24a-*pta* and BL21(DE3)/pET24a-*pta*1.

Genetic manipulations were performed in mutant alleles by using a two-step scarless gene replacement technique employing λ Red recombination [21, 22]. For scarless gene replacement, plasmid pMD-SK was created by cloning two separate PCR products, *kan* and *sacB*, into the plasmid vector pMD18-T. First, *kan* and *sacB* were PCR amplified using plasmid pKD13 and the genomic DNA of *B*. *subtilis* ATCC 6051a as templates, respectively, and the primer pair SK-*kan-sacB* (Table 1). The two PCR products were ligated by overlap extension PCR, then the resulting *kan-sacB* fragment was cloned into pMD18-T, forming pMD-SK. Next, the *kan-sacB* fragment plus homologous sequences was PCR amplified from plasmid pMD-SK using P-*pta* or P-*ackA* primers (Table 1). The underlined letters represent homologous sequences. The single-gene *pta* knockout mutant FB-04(*Δpta*) and single-gene *ackA* knockout mutant FB-04(*ΔAckA*) were constructed by transducing the corresponding *kan-sacB* fragment into cells harboring the helper plasmid pKD46, using kanamycin resistance as the positive selection marker. For the scarless insertion of *pta*1 in the genome of *E*. *coli* FB-04, forming mutant FB-04(*pta*1), the *pta*1 fragment, which was amplified from the *E*. *coli* CCTCC M 2016009 genome using the primer pair V-*pta* (Table 1), was transduced into the *pta* single-gene knockout mutant harboring plasmid pKD46, using 15% sucrose as the negative selection marker. PCR and DNA sequencing were also performed to verify the identities of all alleles and plasmids.

### Media and culture conditions

For enzyme expression, *E*. *coli* strains BL21(DE3)/pET24a-*pta* and BL21(DE3)/pET24a-*pta*1 were grown at 37°C in LB medium. Portions of these seed cultures (10% (v/v)) were used to inoculate 100 mL portions of TB medium supplemented with 30 μg/mL kanamycin in 500 mL flasks at 37°C. After 2 h, isopropyl β-d-thiogalactopyranoside was added to a final concentration of 0.4 mM to induce protein expression. Enzyme expression was performed at 25°C for 24 h in a reciprocal shaker (200 rpm).

For flask cultivation, seed cultures of the L-tryptophan-producing strain FB-04 and its derivatives were grown in LB medium for 10 h at 37°C in a rotary shaker. Aliquots (200 μL) of these seed cultures were used to inoculate 100 mL portions of flask fermentation medium containing (per liter): 24 g K_2_HPO_4_, 9.6 g KH_2_PO_4_, 1g MgSO_4_·7H_2_O, 5 g (NH_4_)_2_SO_4_, 10 g glucose, 15 g yeast, 2 g citric acid, and 3 ml trace element solution. The ingredients of trace element solution have been described elsewhere [5]. These portions of fermentation medium, which were supplemented with 30 μg/mL kanamycin, were incubated in 500 mL flasks at 37°C for 48 h. During the fermentation, NH_4_OH and 10 g/L glucose were added to the medium every 8 h to maintain the pH at 6.5–7.2 and supply a source of carbon, respectively. For metabolomic analysis, cells were collected at 26 h.

For fermentor cultivations, 100 mL seed cultures of FB-04 and its derivatives were first prepared in 500 mL flasks in flask fermentation medium for 10 h at 37°C in a rotary shaker, then transferred to a 3 L BioFlo 110 fermentor (New Brunswick Scientific, USA) containing 900 mL fed-batch fermentation medium containing (per liter) (pH 6.5): 15 g K_2_HPO_4_, 2g MgSO_4_·7H_2_O, 1.6 g (NH_4_)_2_SO_4_, 2 g yeast, 7.5 g glucose, 2 g citric acid, 0.0129 g CaCl_2_, 0.075 g FeSO_4_·7H_2_O. The fed-batch was performed for 54 h, and samples were taken every 3 h. The pH was maintained at 6.5 by automatically adding NH_4_OH. The temperature was kept at 37°C. The dissolved oxygen level was controlled at 20% by adjusting agitation speed. When glucose in the medium was exhausted, glucose (800 g/L) was introduced using an exponential feeding program [23], with cell growth controlled at a specific growth rate of 0.15 h^-1^. All fermentation processes were repeated three times. Fermentation parameters are reported as the mean ± standard deviation of these three replicates.

### Purification of wild-type Pta and its mutant Pta1

Extraction and purification of phosphate acetyltransferase were performed as previously described [24], with slight modification. All operations were performed at 4°C. *E*. *coli* cells harvested from expression cultures were suspended in 20 mM KHCO_3_, then disrupted by sonication for 15 min on ice. Cellular debris was removed by centrifugation at 12,000 × *g* for 30 min. At this point, saturated (NH_4_)_2_SO_4_ was added slowly to the supernatant to attain a final concentration of 50% saturation. The precipitates were stirred overnight at 4°C, collected by centrifugation, then suspended in 50 mM Tris-HCl (pH 8). This solution was dialyzed overnight against 0.2 mM ammonium sulfate in 10 mM Tris-HCl (pH 7.6). Protein purification was performed using a fast protein liquid chromatography system (AKTA FPLC system; GE Healthcare) equipped with a DEAE-Sepharose anion-exchange column (160 mm by 10 mm; Pharmacia Biotech). The dialyzed enzyme solution was diluted 4 times with 10 mM Tris-HCl (pH 8.5), and then loaded onto the DEAE-Sepharose column, which had been pre-equilibrated with 0.05 M ammonium sulfate in 10 mM Tris-HCl (pH 7.8). A linear gradient of 0.05 to 0.2 M ammonium sulfate in 10 mM Tris-HCl (pH 7.8) was used to elute the adsorbed proteins. Fractions containing enzyme were pooled and stored at -20°C.

### Analytical methods

The purified phosphate acetyltransferase was analyzed by SDS-PAGE, and the Bradford method was used to determine protein concentration [25]. Phosphate acetyltransferase activity was measured as previously described [26]. The kinetic parameters (*K*_*m*_, *V*_*max*,_ and *k*_*cat*_ values) of the purified phosphate acetyltransferase were determined by using acetyl-CoA as the substrate (at concentrations of 20, 40, 80, 125, 150, 200, 250, 300, 360, and 400 μmol/L) at 25°C in 100 mM phosphate buffer (pH 7.4). *K*_*m*_ and *V*_*max*_ values were estimated by fitting the initial rate data to the Michaelis-Menten equation using nonlinear regression with GraphPad Prism software (GraphPad Software Inc., San Diego, CA). Enzyme assays were repeated three times. Kinetic data are reported as the mean ± standard deviation of these three replicates.

The optical density at 600 nm (OD_600_) was measured after appropriate dilution. For detection of extracellular metabolites, the supernatant obtained after centrifugation of the fermentation broth was stored at -20°C until analyzed using high performance liquid chromatography (HPLC). The concentrations of glucose and acetate were determined using HPLC with an Aminex HPX-87H column (300 mm × 7.8 mm; Bio-Rid, Hercules, CA) at 50°C, 50 mM H_2_SO_4_ as the mobile phase and a flow rate of 0.5 mL/min. The concentration of L-tryptophan was determined using the method described previously [5], with an Agilent Eclipse XDB-C18 column (15 mm × 4.6 mm).

### Metabolome analysis by GC-MS

Gas chromatography-mass spectrometry (GC-MS) was used to characterize the intracellular metabolite profiles of FB-04, FB-04(*Δpta*) and FB-04(*pta*1). Five biomass samples of each strain were taken manually from the shaker, quickly filtered through a nitrocellulose filter (pore size 45 μm, Pall Corporation), and then immediately quenched in liquid nitrogen. Cells (100 mg wet weight) were suspended in 1.1 ml 90% methanol (-40°C, v/v) and mixed quickly. The cell suspension was frozen and thawed three times, sonicated for 15 min at 4°C, and then held at -20°C for 1 h. After centrifugation at 14,000 × *g* and 4°C for 15 min, isotopically enriched internal standard (L-phenylalanine-^13^C_9_-^15^N) was added into the supernatant to a final concentration of 10 μg/mL, then this mixture was dried using a moderate stream of nitrogen. Methoxylamine hydrochloride in pyridine (20 mg/mL; 30 μL) was added to the powder, and then 30 μL of N,O-Bis(trimethylsilyl)trifluoroacetamide containing 1% trimethylchlorosilane was added to the mixture. Derivatization was allowed to proceed at 70°C for 60 min.

The GC/MS analysis was performed as previously described [27], with slight modifications. The oven temperature was initially held at 70°C for 2 min, then ramped to 160°C at a rate of 6°C/min, to 240°C at a rate of 10°C/min, to 300°C at a rate of 20°C/min, and finally held at 300°C for 8 min. AMDIS software from NIST (National Institute of Standards and Technology) was employed to deconvolute the mass spectra obtained from the raw GC/MS data. The deconvoluted mass spectra were automatically matched with a standard library as previously described that included retention time and mass spectra [27]. Unmatched peaks were analyzed using NIST MS 2.0 software, which automatically searches for compound information from the NIST 11 library and the Golm Metabolome Database. The final data were subjected to principal component analysis using commercial software Simca-P 11.0 (Umetrics AB, Umeå, Sweden) to holistically observe the general clustering and trends among all samples.

## Results

### Sequence comparison of genes involved in the formation of acetate in *E*. *coli* FB-04 and *E*. *coli* CCTCC M 2016009

When they are grown under the same fermentation conditions, less acetate is secreted by *E*. *coli* CCTCC M 2016009 than by *E*. *coli* FB-04 (data not shown). To address this issue, genes involved in acetate formation were analyzed. The *pta*, *ackA*, and *poxB g*enes from *E*. *coli* FB-04 and the *pta*1, *ackA*1, and *poxB*1 genes from *E*. *coli* CCTCC M 2016009 were amplified using PCR and then sequenced. Comparison of the three pairs of genes (*pta/pta*1, *ackA/ackA*1, *poxB/poxB*1) revealed a single base difference (c206t) between *pta* and *pta*1; the sequences of *ackA* and *ackA*1 were identical, as were those of *poxB* and *poxB*1. This difference leads to a single amino acid substitution (Pro69 to Leu) in Pta1, compared with Pta.

### Enzymatic analysis of Pta and Pta1

**Fig 1:**
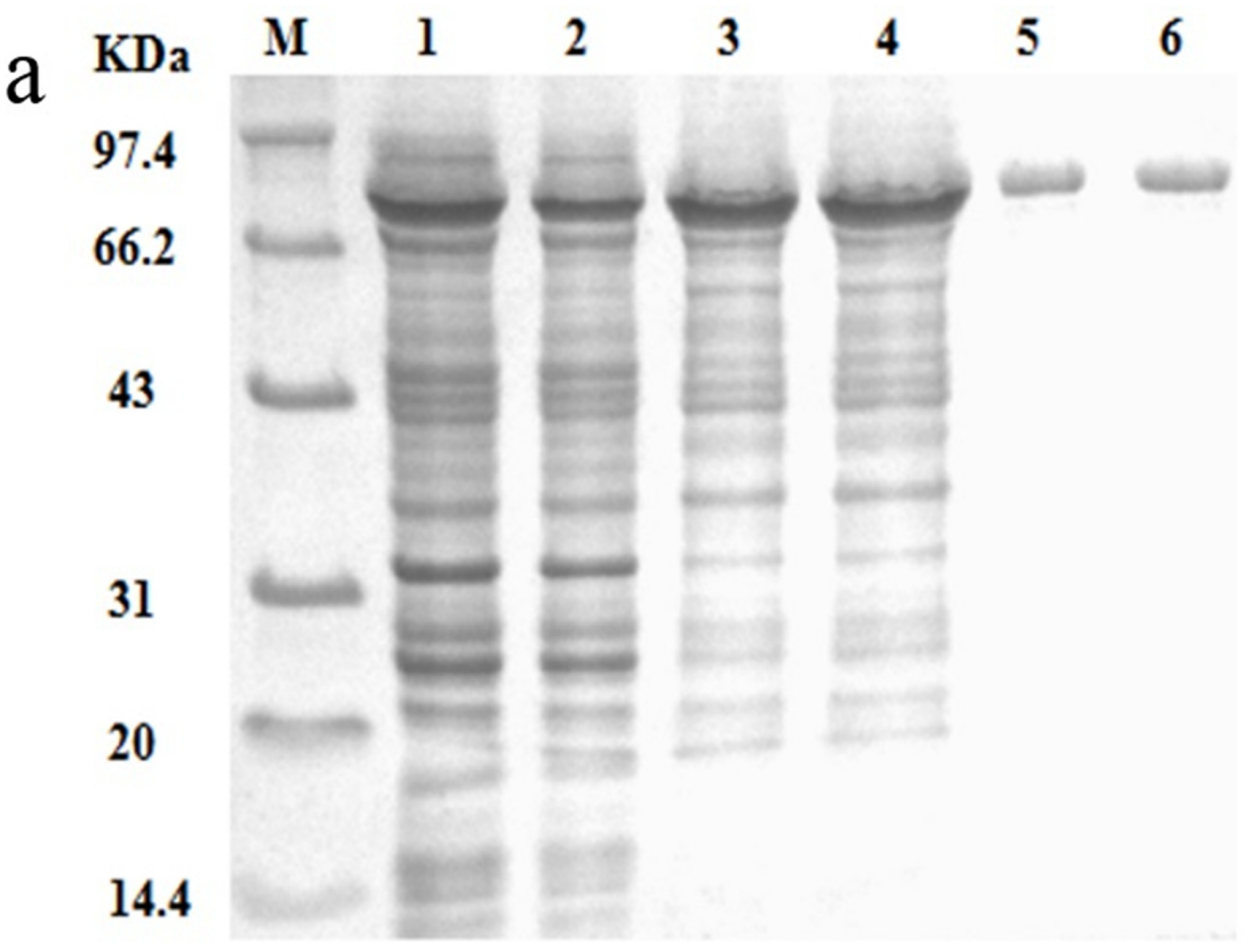
SDS-PAGE analysis of enzymes. (a) SDS-PAGE analysis of Pta and Pta1 followed by the protein concentration. Lanes contain: cell lysates from expression cultures of *E*. *coli* BL21(DE3)/pET24a-*pta* (1.2 mg/mL) (lane 1) and *E*. *coli* BL21(DE3)/pET24a-*pta*1 (1.1 mg/mL) (lane 2); 50% saturated (NH_4_)_2_SO_4_ precipitations of Pta (0.7 mg/mL) (lane 3) and Pta1(0.8 mg/mL) (lane 4); Purified enzymes: Pta (0.2 mg/mL) (lane 5) and (0.2 mg/mL)Pta1 (lane 6); and protein molecular weight markers (lane M).

To evaluate the activities of Pta and Pta1, the two enzymes were overexpressed in *E*. *coli* BL21(DE3). SDS-PAGE analysis of cell lysates revealed protein bands at approximately 77 kDa, which correspond to those observed with purified recombinant Pta and Pta1(Fig 1). The positions of these bands are consistent with their predicted molecular masses. The protein concentrations were almost the same, indicating that the difference between the expression levels of Pta and Pta1 was not significant. Purified Pta and Pta1 were subjected to kinetic analysis. The catalytic constant (*k*_*cat*_) of Pta1 was 38% lower than that determined for Pta, while its *K*_m_ was 190% higher than that of Pta. Thus, the *k*_*cat*_ /*K*_*m*_ value of Pta1 was only about 21% that of Pta (Table 2).

**Table 2:** Kinetic parameters for Pta and Pta1.

Enzyme	*k*_*cat*_ (s^-1^)	*K*_*m*_ (μM)	*k*_*cat*_ /*K*_*m*_ (μM^-1^ s^-1^)
Pta	2,380 ±120	89 ± 6.3	27 ± 3.6
Pta1	1,480 ± 74	260 ±13	5.7 ± 0.5

All data are the average (± standard deviation) of three independent experiments.

### Effect of *pta*1 genomic substitution on acetate formation and L-tryptophan production

**Fig 2:**
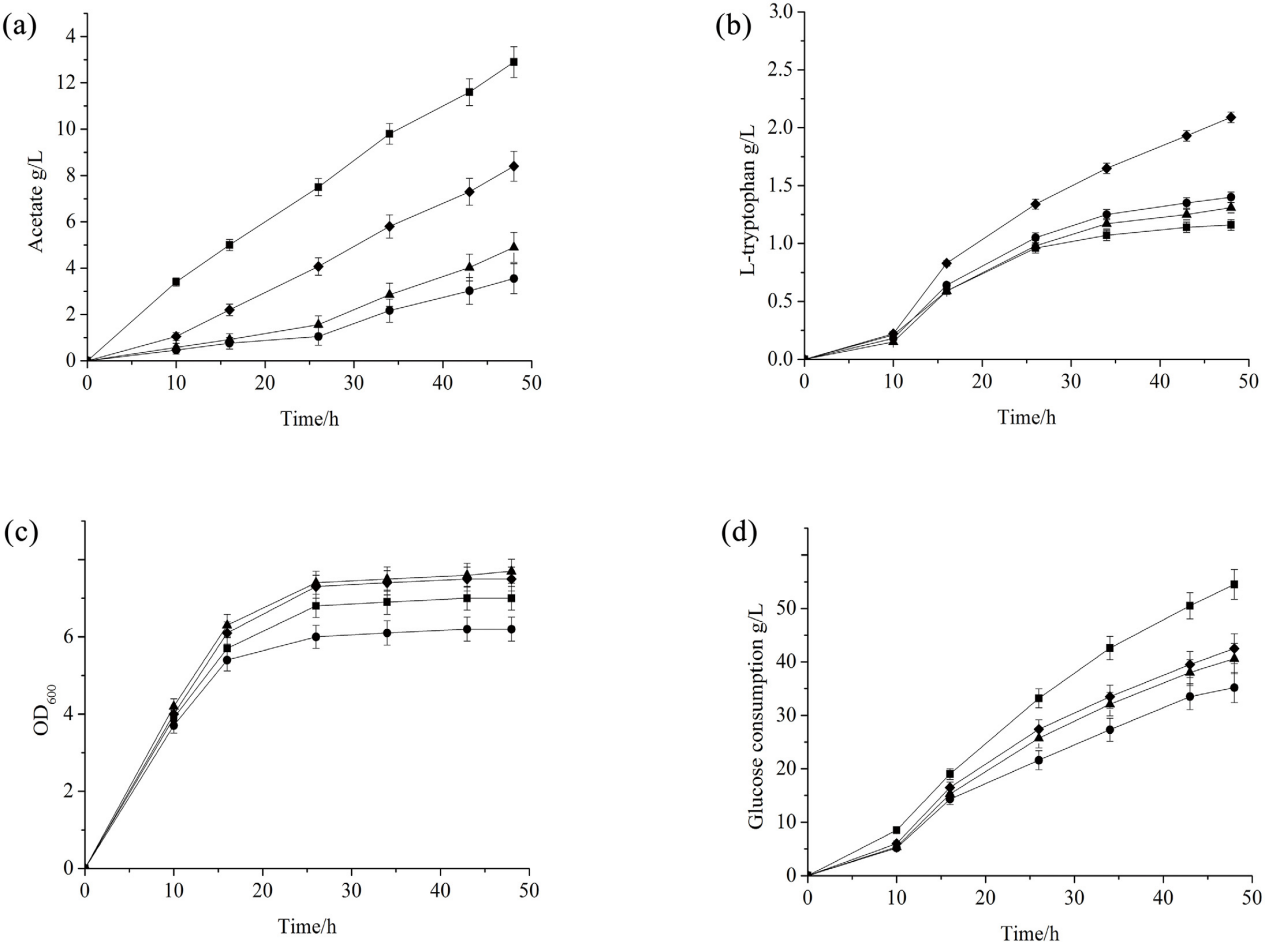
Flask cultivation of different strains. (a) Acetate levels; (b) L-tryptophan levels; (c) biomass levels; (d) glucose consumption levels. FB-04 (square); FB-04(*Δpta*) (circle); FB-04(*ΔackA*) **(**triangle); FB-04(*pta*1) (diamond).

To investigate the effect of Pta-AckA pathway alteration on acetate formation, *E*. *coli* strain FB-04(*Δpta*), in which *pta* has been deleted, FB-04(*ΔackA*), in which *ackA* has been deleted, and FB-04(*pta*1), in which *pta* has been replaced by *pta*1, were constructed from *E*. *coli* FB-04 using λ Red recombination. Then, the fermentation performances of FB-04(*Δpta*), FB-04(*ΔackA*) and FB-04(*pta*1) were compared with that of parent strain FB-04 in a series of shake-flask cultures. Throughout the entire fermentation processes, the acetate levels in FB-04(*Δpta*), FB-04(*ΔackA*) and FB-04(*pta*1) cultures were lower than that in cultures of their parent strain FB-04 (Fig 2a). Fermentation parameters for different strains are summarized in Table 3. Strains FB-04(*Δpta*), FB-04(*ΔackA*) and FB-04(*pta*1) showed remarkable decreases in acetate formation, corresponding to 72%, 62%, and 35% that of the parent strain FB-04, respectively.

**Table 3:** Comparison of fermentation parameters for different strains in flask cultivation and fed-batch fermentation.

Strain	Glucose	Maximum	Maximum	L-tryptophan	L-tryptophan	Maximum
	consumption	biomass	L-tryptophan	productivity	yield	acetate
	(g/L)	(OD_600_)	(g/L)	(g/L/h)	per glucose (g/g)	(g/L)
Flask fermentation						
FB-04	54.5±5.0	7.0±0.4	1.1±0.1	0.020±0.002	0.020±0.003	12.9±0.7
FB-04(*Δpta*)	35.2±2.5	6.2±0.3	1.4±0.1	0.029±0.002	0.040±0.005	3.6±0.2
FB-04(*ΔackA*)	40.6±4.1	7.7±0.6	1.3±0.1	0.027±0.001	0.032±0.003	4.9±0.3
FB-04(*pta*1)	42.5±4.3	7.5±0.6	2.1±0.2	0.044±0.005	0.049±0.003	8.4±0.5
Fed-batch fermentation						
FB-04	357±22	73.6±0.6	38.1±2.3	0.70±0.04	0.11±0.01	4.3±0.3
FB-04(*Δpta*)	292±19	52.5±3.8	21.8±1.5	0.40±0.03	0.08±0.01	0.5±0.3
FB-04(*ΔackA*)	338±20	76.8±5.8	40.2±2.0	0.74±0.04	0.12±0.01	1.5±0.2
FB-04(*pta*1)	346±27	82.8±6.5	44.0±2.8	0.82±0.06	0.13±0.01	2.1±0.3

All data are the average (with standard deviation) of three independent experiments.

L-tryptophan production levels were also determined (Fig 2b). The L-tryptophan titer of strain FB-04(*pta*1) (2.1 g/L) was significantly higher than that of FB-04 (1.1 g/L), FB-04(*Δpta*) (1.4 g/L) and FB-04(*ΔackA*) (1.3 g/L), corresponding to increases of 91%, 50%, and 62%, respectively (p < 0.01, Student’s *t*-test). As shown in Fig 2c, the growth of FB-04(*Δpta*) was restricted, while FB-04(*ΔackA*), FB-04(*pta*1) and FB-04 displayed similar growth curves. The final biomass level (OD_600_) of FB-04(*Δpta*) was 11% less than that of FB-04, while the final biomass levels of FB-04(*ΔackA*) and FB-04(*pta*1) were slightly higher (Table 3). During cell growth, glucose consumption increased with time for all strains (Fig 2d). The glucose consumption rate of FB-04(*pta*1) was lower than that of FB-04, while the glucose consumption rate of FB-04(*Δpta*) was lowest. In addition, FB-04(*pta*1) displayed L-tryptophan productivity and L-tryptophan yield per glucose that were significantly greater than those of FB-04, FB-04(*ΔackA*), and FB-04(*Δpta*) (p<0.01, Student’s *t*-test) (Table 3).

### Fed-batch fermentation of FB-04(*pta*1)

**Fig 3:**
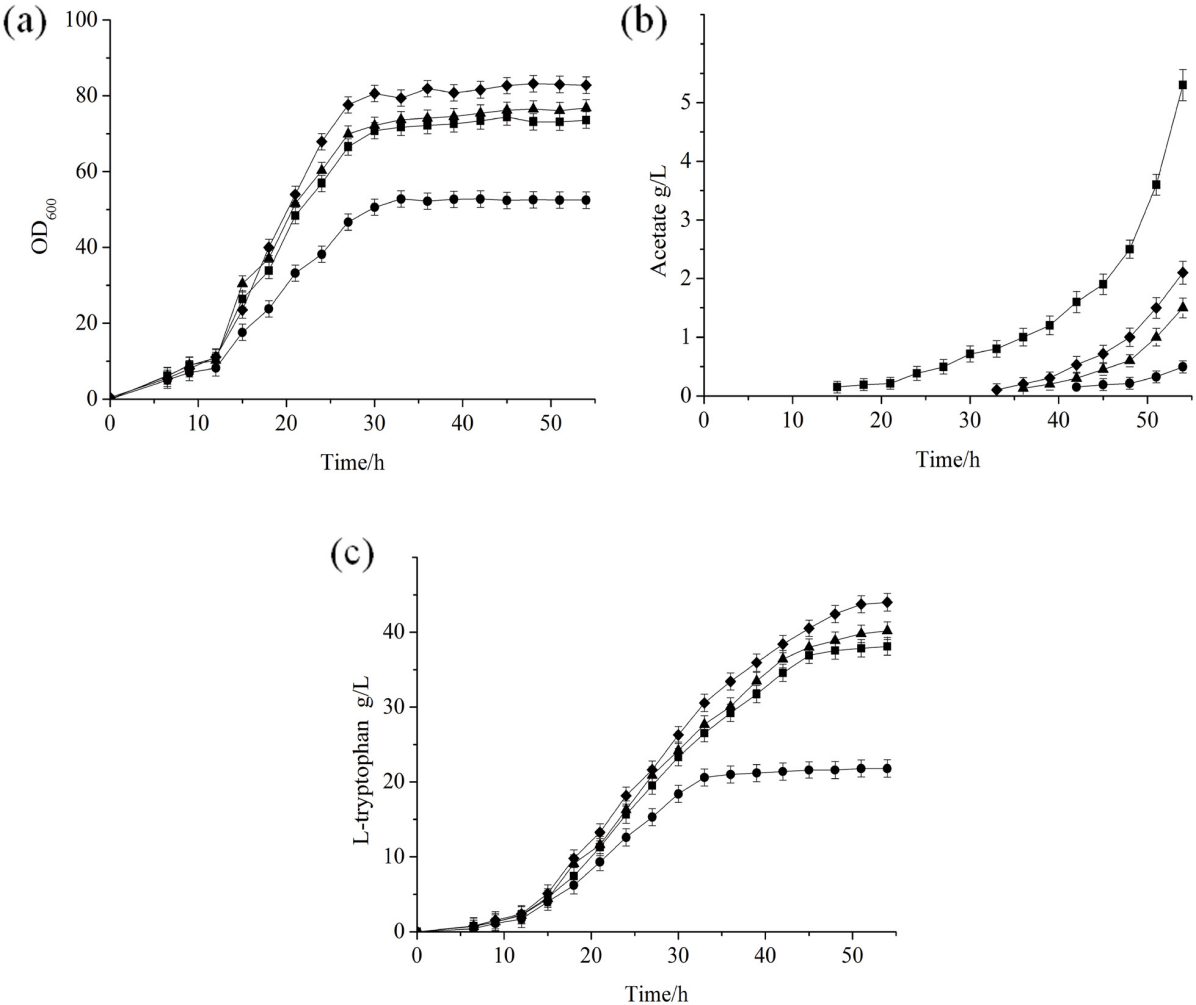
Fed-batch fermentation of different strains. (a) Biomass levels; (b) Acetate levels; (c) L-tryptophan levels. FB-04 (square); FB-04(*Δpta*) (circle); FB-04(*ΔackA*) **(**triangle); FB-04(*pta*1) (diamond).

Since FB-04(*pta*1) produced more L-tryptophan, it was further evaluated in fed-batch fermentation, comparing its performance with those of FB-04, FB-04(*ΔackA*) and FB-04(*Δpta*) (Fig 3). The growth of FB-04(*Δpta*) was impaired, while the growth curves for FB-04, FB-04(*ΔackA*) and FB-04(*pta*1) were similar, although FB-04(*pta*1) achieved the highest total biomass (Fig 3a). Levels of acetate produced by all strains are shown in Fig 3b. The acetate content of FB-04 was undetectable during the first 14 h, and then increased with time, reaching about 2.0 g/L at 27 h and 4.3 g/L at the end of fermentation. The acetate levels of FB-04(*Δpta*) and FB-04(*ΔackA*) were extremely low throughout the fermentation processes. In contrast, the acetate content of FB-04(*pta*1) began to increase noticeably at 32 h and reached a final concentration of 2.1 g/L at the end of fermentation.

L-tryptophan production by all strains increased over the first 30 h of fermentation processes (Fig 3c). The L-tryptophan yield of FB-04 continued to increase during the subsequent 14 h, but remained almost unchanged after the acetate content reached about 2.0 g/L. The L-tryptophan yield of FB-04(*Δpta*) was lower than those of FB-04, FB-04(*ΔackA*) and FB-04(*pta*1) throughout the entire fermentation processes. It reached its maximum at 30 h despite the fact that the acetate concentration was still extremely low. L-tryptophan yields of FB-04(*ΔackA*) and FB-04(*pta*1) also increased with time, while FB-04(*pta*1) achieved a more significant increase in L-tryptophan production. The final L-tryptophan titer of FB-04(*pta*1) (44 g/L) was significantly greater than those of FB-04, FB-04(*ΔackA*) and FB-04(*Δpta*), corresponding to increases of 15%, 9.5% and 100%, respectively (p < 0.01, Student’s *t*-test) (Table 3). The L-tryptophan yield per glucose of FB-04(*pta*1) was also significantly greater than those of FB-04, FB-04(*ΔackA*) and FB-04(*Δpta*) (p < 0.01, Student’s *t*-test) (Table 3).

### The principal component differences caused by genetic modification

**Fig 4:**
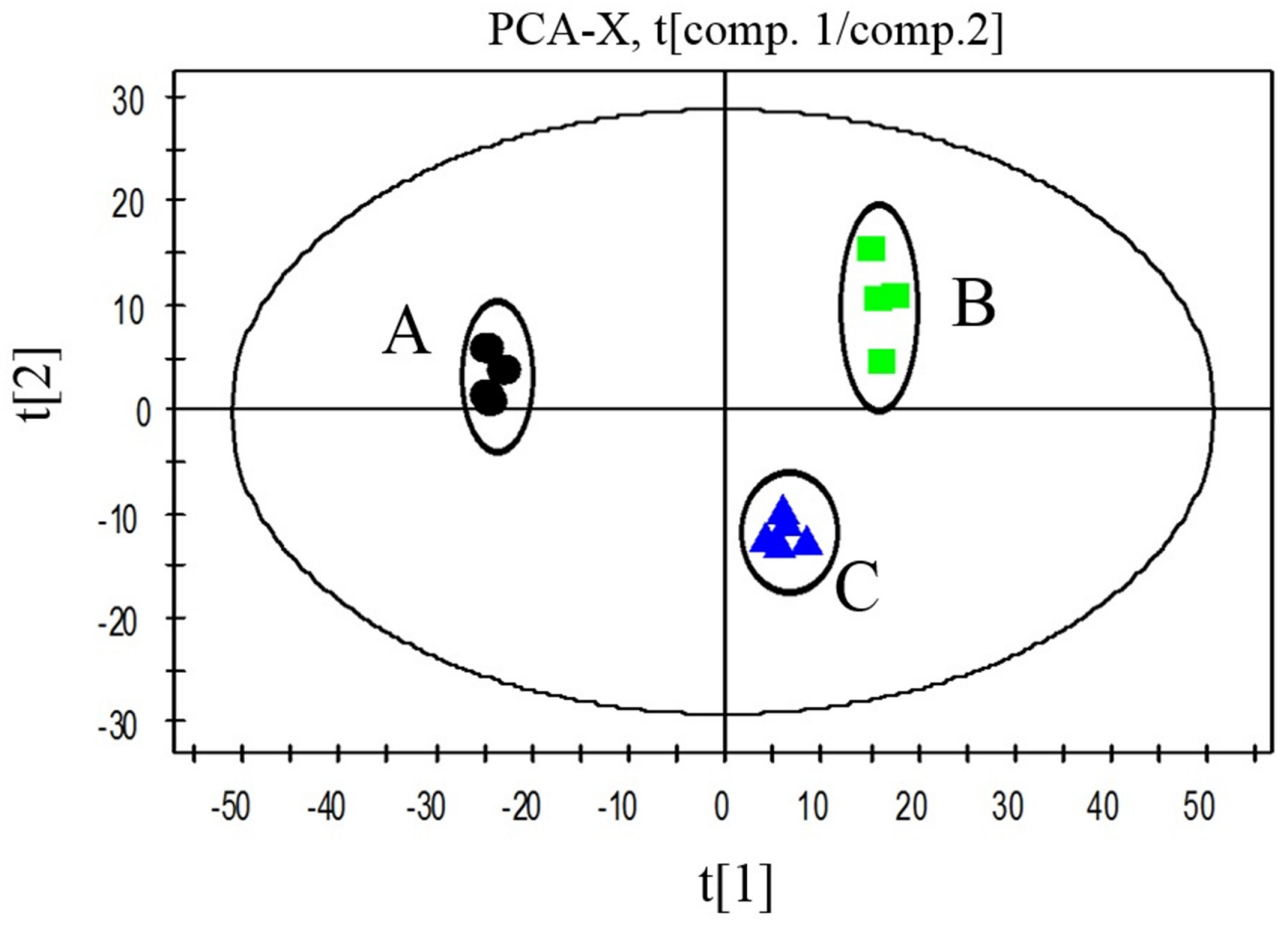
PCA of FB-04, FB-04(*Δpta*), and FB-04(*pta*1). The x axis represents the first principal component, PC1 (t[1]), which explains the major differences; the y axis represents the second principal component, PC2 (t[2]), which explains the minor differences. A, FB-04; B, FB-04(*Δpta*); C, FB-04(*pta*1)

Principal component analysis (PCA) was carried out to gain insight into the multivariate data and evaluate biological alteration. Clustering of biological samples was based on their similarities and differences in the metabolite dataset. In the PCA score plot, each data point reflects a linear combination of the total metabolites from each sample. The distances between the groups give a measure of the overall variation among the metabolic profiles of the different strains. As shown in Fig 4, three groups of FB-04, FB-04(*Δpta*) and FB-04(*pta*1) were distributed in different areas of the PCA score plot.

### Changes of carbon flux in strain carrying a *pta* deletion or a *pta*1 genomic substitution

In the present study, more than 80 intracellular metabolites that showed different levels in FB-04, FB-04(*Δpta*) and FB-04(*pta*1) were identified using GC-MS. Of these 80 metabolites, 23 of them were involved in L-tryptophan biosynthesis. To identify the potential reasons for the significant diversity in metabolic profiles caused by genetic modification, the relative levels of the 19 intracellular metabolites were determined (see S1 Table).

**Fig 5:**
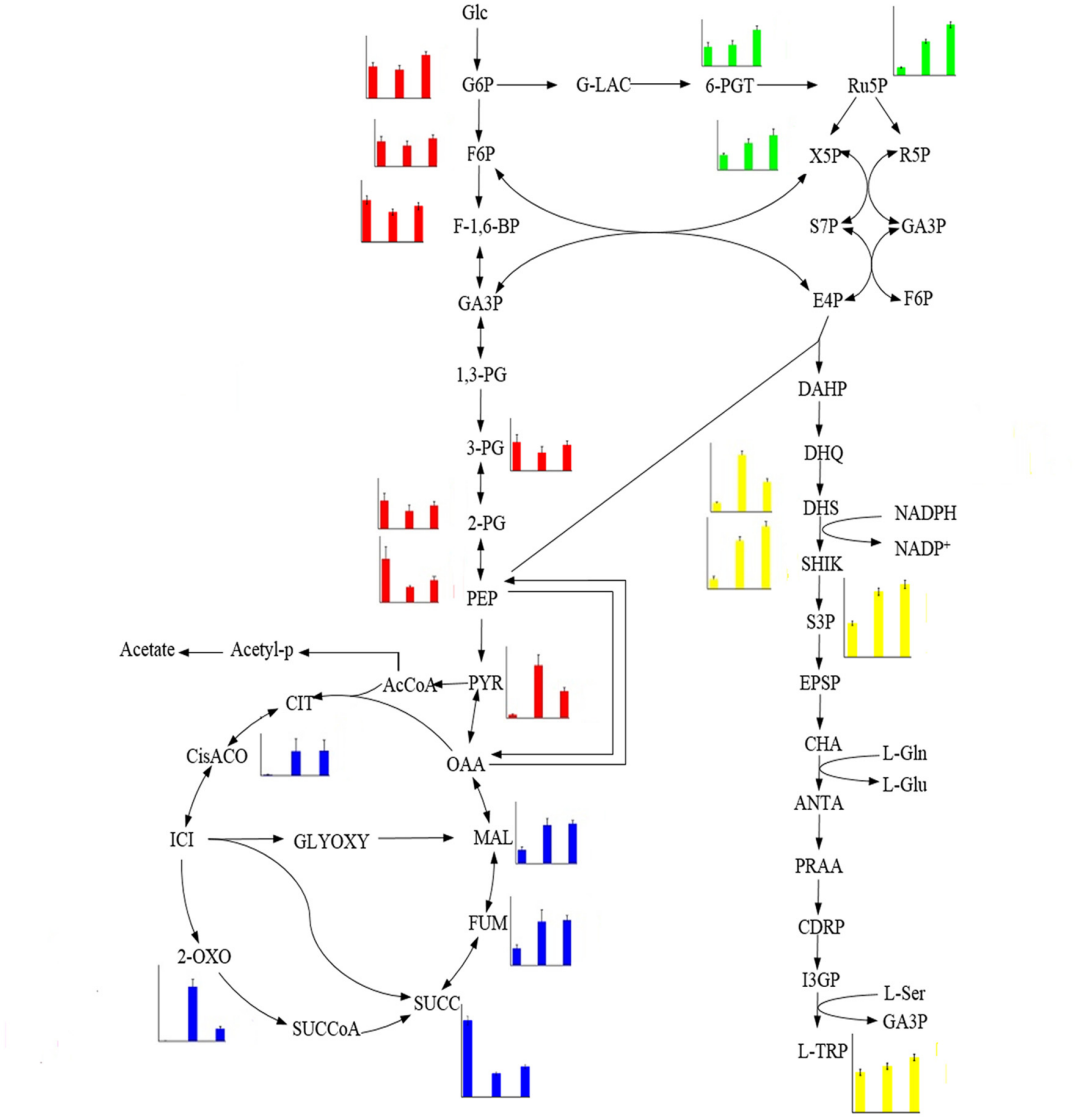
Levels of intermediates involved in L-tryptophan biosynthesis detected in FB-04, FB-04(*Δpta*), and FB-04(*pta*1). The strains along the x-axes are FB-04, FB-04(*Δpta*), and FB-04(*pta*1), successively. The y-axes reflect the relative abundance of each intermediate, which was calculated by normalization of the peak area of each metabolite against total peak area within sample. Glc, glucose; G6P, glucose-6-phosphate; F6P, fructose-6-phosphate; 1,6-BP, fructose-1,6-bisphosphate; GA3P, glyceraldehyde-3-phosphate; 3-PG, 3-phosphoglycerate; 2-PG, 2-phosphoglycerate; PEP, phosphoenolpyruvate; PYR, pyruvate; AcCoA, acetyl coenzyme A; CIT, citrate; ICI, isocitrate; 2-OXO, 2-oxoglutarate; SUCCoA, succinyl coenzyme A; SUCC, succinate; FUM, fumarate; MAL, malate; OAA, oxaloacetate; G-LAC, 6-phosphoglucono-1,5-lactone; 6-PGT, 6-phosphogluconate; Ru5P, ribulose-5-phosphate; X5P, xylulose-5-phosphate; R5P, ribose-5-phosphate; S7P, sedoheptulose-7-phosphate; E4P, erythrose- 4-phosphate; DAHP, 3-deoxy-d-arabinoheptulosonate-7-phosphate; DHQ, 3-dehydroquinate; Quin, quinone; DHS, 3-dehydroshikimate; SHIK, shikimate; S3P, shikimate-3-phosphate; EPSP, 5-enolpyruvoylshikimate; CHA; chorismate; ANTA, anthranilate; PRAA, phosphoribosyl anthranilate; CDRP, 1-(o-carboxyphenylamino)-1-deoxyribulose-5-phosphate; I3GP, indole 3-glycerolphosphate; L-Trp, L-tryptophan; Acetyl-p, acetyl phosphate; GLYOXY, glyoxylate pathway.

The levels of intracellular intermediates in the three strains FB-04, FB-04(*Δpta*) and FB-04(*pta*1) are shown in Fig 5. Interestingly, metabolic intermediate levels in these three strains changed remarkably. Comparing FB-04(*Δpta*) with the parent strain FB-04, the overall levels of glycolysis intermediates were reduced; however, pyruvate accumulated significantly. Moreover, the overall levels of TCA cycle intermediates and pentose phosphate pathway intermediates were increased. Notably, levels of common aromatic pathway intermediates, such as 3-dehydroshikimate, shikimate and shikimate-3-phosphate, were increased in FB-04(*Δpta*).

Compared with FB-04, FB-04(*pta*1) displayed a metabolic performance similar to that of FB-04(*Δpta*). The overall levels of glycolysis intermediates were slightly decreased, while the overall levels of pentose phosphate pathway intermediates, TCA cycle intermediates, and common aromatic pathway intermediates were significantly increased. Moreover, compared with FB-04(*Δpta*), the overall levels of intermediates in glycolysis and the pentose phosphate pathway were increased in FB-04(*pta*1), while levels of TCA cycle intermediates were reduced. In addition, the levels of shikimate and shikimate-3-phosphate, were higher in FB-04(*pta*1), although that of 3-dehydroshikimate was reduced.

## Discussion

L-tryptophan is mainly produced by microbial fermentation using *Escherichia coli* or *Corynebacterium glutamicum*. A randomly mutagenized *E*.*coli* strain was shown to produce up to 54.6 g/L L-tryptophan when fed L-tryptophan precursors [6]. With the recent advances in molecular technology, several studies have been conducted in an effort to construct L-tryptophan-producing strains with defined genetic modifications [15, 28–30]. For example, genetic modification of a classically derived L-tryptophan-producing *Corynebacterium glutamicum* strain increased L-tryptophan production to 58 g/L [28]. *E*.*coli* strain D*pta*/*mtr*-Y, developed by Wang et al. [15], achieved an L-tryptophan yield of 48.68 g/L.

In this study, mutant strains FB-04(*Δpta*) and FB-04(*ΔackA*) were constructed to decrease acetate accumulation. Deletion of *pta* or *ackA* led to substantially lowered acetate formation (Table 3). Pta plays a more important role in the Pta-AckA pathway, in view of the fermentation performance of FB-04(*Δpta*) and FB-04(*ΔackA*) (Figs 2a and 3b). Reduced acetate levels were conducive to L-tryptophan biosynthesis, as improved L-tryptophan titers were observed in FB-04(*Δpta*) and FB-04(*ΔackA*), compared with FB-04 (Fig 2b). Notably, deletion of *pta* achieved a more significant increase in L-tryptophan production than deletion of *ackA* in shake-flask fermentations (Fig 2b). However, FB-04(*Δpta*) exhibited seriously restricted growth, which was consistent with previous findings [14, 17].

To avoid the physiological defects caused by *pta* deletion, we identified a mutant Pta (Pta1) from *E*. *coli* CCTCC M 2016009. Kinetic analysis showed that the *K*_m_ of Pta1 was 190% higher than that of Pta, and the *k*_*cat*_ /*K*_*m*_ value of Pta1 was only about 21% that of Pta (Table 2). These data indicate that Pta1 possesses lower catalytic activity and substrate binding affinity. In this study, we constructed FB-04(*pta*1), in which *pta* was replaced with *pta*1. This substitution not only resulted in a noticeably lower ability to secrete acetate, it also reversed the growth defect caused by *pta* deletion (Fig 3a and 3b). It has been reported that the growth defect caused by *pta* deletion results from disturbing acetyl-CoA flux, and that any method that relieves the oversupply of acetyl-CoA would compensate for this defect [17]. The *pta*1 genomic substitution might alleviate the accumulation of acetyl-CoA to a certain degree by weakening the Pta-AckA pathway. In addition to its lowered acetate accumulation and normal growth characteristics, strain FB-04(*pta*1) showed a substantial improvement in L-tryptophan yield over those of FB-04 and FB-04(*Δpta*) (Fig 3c).

To investigate the effect of Pta alteration on cells, changes in metabolic flow were explored. Because the deletion of *pta* reduces the flux through glycolysis and increases the flux through the TCA cycle caused by the accumulation of acetyl-coA and pyruvate [17], FB-04(*Δpta*) displayed decreased levels of glycolytic intermediates and increased levels of TCA cycle intermediates (Fig 5). The activity of glucose-6 phosphate dehydrogenase, the first enzyme in the pentose phosphate pathway, is known to be upregulated by *pta* deletion [31]. This contributes to the increased flux through the pentose phosphate pathway, which is consistent with the increased levels of pentose phosphate pathway intermediates seen in this study (Fig 5). The metabolic performance of FB-04(*pta*1) differed from that of FB-04 in ways similar to the metabolic differences between FB-04 and FB-04(*Δpta*). FB-04(*pta*1) displayed decreased levels of glycolytic intermediates and increased levels of TCA cycle and pentose phosphate pathway intermediates (Fig 5). In addition, the levels of pentose phosphate pathway intermediates in FB-04(*pta*1) were greater than those of FB-04(*Δpta*)). This difference may be ascribed to the more reasonable distribution of metabolic flow among the central metabolic pathways caused by the *pta*1 genomic substitution. Increased flux through the pentose phosphate pathway not only supplies additional erythrose 4-phosphate, an important L-tryptophan precursor, it also allows the full utilization of another precursor, phosphoenolpyruvate, which boosts carbon flux through the common aromatic pathway [32]. This is consistent with the increased levels of common aromatic pathway intermediates in FB-04(*pta*1) (Fig 5). The level of 3-dehydroshikimate, an intermediate in the common aromatic pathway, was lower in FB-04(*pta*1) than in FB-04(*Δpta*) (Fig 5). The improved metabolic flow in the pentose phosphate pathway caused by the *pta*1 genomic substitution could increase the formation of NADPH, an essential cofactor in the common aromatic pathway [32]. This would drive carbon flux from 3-dehydroshikimate to shikimate, decreasing the level of 3-dehydroshikimate.

In view of the length of the L-tryptophan biosynthetic pathway and its complicated regulation mechanism, relying only on decreased acetate production and changes in metabolism caused by the *pta*1 genomic substitution would not achieve a remarkable improvement in L-tryptophan production. The metabolic alterations caused by the *pta*1 genomic substitution also increased levels of L-tryptophan precursors, which further contributed to L-tryptophan biosynthesis. In future studies, overexpression of the genes *ppsA* and *tktA*, which are involved in the biosynthesis of phosphoenolpyruvate and erythrose 4-phosphate, respectively [32], may significantly improve L-tryptophan production.

## Conclusion

A *pta* gene knockout was constructed in *E*. *coli* FB-04 using λRed recombination to reduce the formation of acetate, but the resulting strain (*E*. *coli* FB-04(*Δpta*)) exhibited a growth defect. Then, a mutant (Pta1) that exhibits lower catalytic capacity and substrate affinity than Pta because of a single substitution (Pro69Leu) was identified in *E*. *coli* CCTCC M 2016009. This variant (*pta*1), was used to replace the *pta* gene of *E*. *coli* FB-04, forming strain FB-04(*pta*1). FB-04(*pta*1) not only lacked the growth defect of FB-04(*Δpta*) and showed improved fermentation performance, it also displayed a 91% increase in L-tryptophan yield during flask fermentation, compared with FB-04, while acetate production decreased by 35%. Moreover, acetate secretion by FB-04(*pta*1) was slower than that by FB-04 throughout the fed-batch fermentation processes, and finally the L-tryptophan yield of FB-04(*pta*1) represented a 15% increase over that of FB-04. Metabolomics analysis showed that the *pta*1 genomic substitution slightly decreased carbon flux through glycolysis and significantly increased carbon flux through the pentose phosphate and common aromatic pathways, contributing to the biosynthesis of L-tryptophan.

## Supporting Information

S1 Table The levels of intracellular metabolites involved in L-tryptophan biosynthesis in different strain. To ensure the accuracy of the data, five biomass samples of each strain were subjected to GC-MS. Most of the samples were within 95% confidence interval, except samples A1 and B1 (date not show). A1 and B1 were judged to be abnormal samples and were omitted from the analysis to ensure the reliability of the results. The rt_mz values express the mass-to-charge ratio of chromatographic retention time.
(DOC)
