# Supplementary material for: L-Tryptophan Production in Escherichia coli Improved by Weakening the Pta-AckA Pathway
Source: PLoS One. 2016 Jun 27;11(6):e0158200. doi: 10.1371/journal.pone.0158200 (PMC4922666; doi:10.1371/journal.pone.0158200)
Supplement: S1 Table — To ensure the accuracy of the data, five biomass samples of each strain were subjected to GC-MS. Most of the samples were within 95% confidence interval, except samples A1 and B1 (date not show). A1 and B1 were judged to be abnormal samples and were omitted from the analysis to ensure the reliability of the results. The rt_mz values express the mass-to-charge ratio of chromatographic retention time. (DOC) [file pone.0158200.s001.doc]

| 25.57_202 | 25.64_299 | 21.42_204 | 21.21_296 | 24.55_315 | 24.65_357 | 26.93_387 | 20.96_156 | 18.76_246 | 16.29_233 | 13.38_245 | 12.63_247 | 18_198 | 21.5_273 | 13.56_204 | 6.59_174 | 18.36_369 | 20.99_369 | 21.34_357 | 28.4_315 | 26.2_315 | 26.5_387 | rt_mz  * |
| --- | --- | --- | --- | --- | --- | --- | --- | --- | --- | --- | --- | --- | --- | --- | --- | --- | --- | --- | --- | --- | --- | --- |
| L-TRP | S-3P | SHIK | DHS | X-5-P | Ru-5-P | 6-PGT | L-GLN | L-GLU | MAL | FUM | SUCC | 2-OXO | CIT | L-Ser | PYR | PEP | 2-PG | 3-PG | F-1,6-BP | F-6-P | G-6-P | metabolite |
| 6.848709 | 0.051799 | 1.752140 | 0.085581 | 0.021015 | 0.002652 | 0.004933 | 0.003952 | 0.342577 | 0.034952 | 0.224184 | 4.177617 | 0.001000 | 0.012982 | 0.022555 | 0.299065 | 0.069111 | 0.02086 | 0.305743 | 0.076928 | 0.045521 | 1.951198 | A2 |
| 7.379191 | 0.050860 | 1.109073 | 0.070404 | 0.022422 | 0.004072 | 0.005590 | 0.004820 | 0.341162 | 0.037665 | 0.256372 | 3.969396 | 0.000900 | 0.016860 | 0.020578 | 0.379184 | 0.038253 | 0.013129 | 0.208036 | 0.084498 | 0.067748 | 2.238458 | A3 |
| 7.078173 | 0.069753 | 1.580768 | 0.075212 | 0.024524 | 0.003355 | 0.004838 | 0.004729 | 0.480102 | 0.032099 | 0.193106 | 3.862710 | 0.000800 | 0.021567 | 0.031044 | 0.323648 | 0.042039 | 0.012480 | 0.173944 | 0.079677 | 0.053242 | 2.055621 | A4 |
| 7.613570 | 0.050034 | 0.963149 | 0.073659 | 0.019030 | 0.003105 | 0.005316 | 0.006366 | 0.578481 | 0.051512 | 0.314124 | 4.607693 | 0.001200 | 0.030961 | 0.023728 | 0.449959 | 0.061231 | 0.018684 | 0.300579 | 0.099151 | 0.045068 | 2.550537 | A5 |
| 7.840549 | 0.076600 | 6.776998 | 0.498221 | 0.031911 | 0.010946 | 0.005511 | 0.076398 | 6.585116 | 0.095681 | 0.603011 | 1.464372 | 0.318800 | 0.407539 | 0.064514 | 6.409196 | 0.018939 | 0.009006 | 0.133927 | 0.043110 | 0.047080 | 2.019946 | B2 |
| 8.346701 | 0.104265 | 7.366663 | 0.494912 | 0.043544 | 0.011167 | 0.006592 | 0.090981 | 5.641814 | 0.119307 | 0.532000 | 1.367925 | 0.273300 | 0.786090 | 0.060188 | 6.894141 | 0.019622 | 0.008814 | 0.134586 | 0.053210 | 0.052372 | 1.993385 | B3 |
| 8.052430 | 0.091406 | 6.745182 | 0.538709 | 0.044702 | 0.009220 | 0.007869 | 0.069673 | 7.658758 | 0.093170 | 0.520939 | 1.247757 | 0.239600 | 0.337605 | 0.057598 | 4.233757 | 0.019481 | 0.015329 | 0.236246 | 0.062585 | 0.046171 | 2.37954 | B4 |
| 8.810121 | 0.103285 | 6.039435 | 0.452935 | 0.035276 | 0.008105 | 0.005171 | 0.099912 | 7.449089 | 0.133293 | 0.889024 | 1.479143 | 0.316000 | 1.006105 | 0.046689 | 6.074858 | 0.015890 | 0.007885 | 0.128729 | 0.036578 | 0.030775 | 1.914009 | B5 |
| 8.442249 | 0.111107 | 7.747604 | 0.268296 | 0.045817 | 0.01064 | 0.011392 | 0.059049 | 4.503738 | 0.11100 | 0.643310 | 1.524387 | 0.07010 | 0.511173 | 0.020634 | 3.218927 | 0.021512 | 0.011844 | 0.1932910 | 0.076266 | 0.059123 | 3.085961 | C1 |
| 9.313930 | 0.101459 | 9.484093 | 0.260928 | 0.044853 | 0.012557 | 0.009308 | 0.068003 | 3.881714 | 0.122298 | 0.572165 | 1.694095 | 0.076200 | 0.814667 | 0.019607 | 3.126054 | 0.024197 | 0.010412 | 0.177494 | 0.074213 | 0.061301 | 2.809701 | C2 |
| 8.762629 | 0.089065 | 9.117986 | 0.300521 | 0.045699 | 0.008873 | 0.010143 | 0.055123 | 4.443290 | 0.108120 | 0.710939 | 1.830621 | 0.045100 | 0.380771 | 0.018747 | 2.367463 | 0.026747 | 0.014966 | 0.260500 | 0.090222 | 0.058461 | 3.215636 | C3 |
| 9.467538 | 0.113670 | 8.891046 | 0.243187 | 0.049115 | 0.016120 | 0.012237 | 0.075483 | 3.853124 | 0.105244 | 0.633800 | 1.475598 | 0.062400 | 0.496826 | 0.019574 | 2.968349 | 0.032834 | 0.015655 | 0.246291 | 0.076681 | 0.068424 | 3.170898 | C4 |
| 8.935666 | 0.087156 | 8.445006 | 0.233019 | 0.066668 | 0.013518 | 0.010305 | 0.100723 | 3.764394 | 0.126381 | 0.735564 | 1.704296 | 0.073200 | 1.044147 | 0.028774 | 3.362803 | 0.029601 | 0.014390 | 0.235098 | 0.062759 | 0.048227 | 2.731503 | C5 |
